# The scientific journal, beyond experts: the communication strategies of The New England Journal of Medicine and The Lancet

**DOI:** 10.1590/S0104-59702026000100005en

**Published:** 2026-05-11

**Authors:** Germana Barata, Carolina Ferreira Medeiros

**Affiliations:** i Researcher, Laboratório de Estudos Avançados em Jornalismo/Núcleo de Desenvolvimento da Criatividade/ Universidade Estadual de Campinas. Campinas – SP – Brazil. germana@unicamp.br; ii Researcher, Instituto de Biologia/Universidade Estadual de Campinas. Campinas – SP – Brazil. carolinafmedeiros07@gmail.com

**Keywords:** Science communication, History of medicine, Medical journals, The Lancet, The New England Journal of Medicine

## Abstract

The health sector pioneered the dialog with society, the media, health professionals and decision-makers, due to great public interest in this area and its social responsibility. Two internationally prestigious journals with two centuries of history (*The New England Journal of Medicine* and *The Lancet*) are examples of communication strategies that extend beyond the walls of academia. This article analyzes the editorial policies, commemorative editorials and communication strategies of these two journals in order to understand their relationship with the public throughout history. This analysis shows that communication to non-experts is an important part of editorial policies, and has expanded over the years in both journals.

## The increasingly important social role of scientific journals

It became clear during the covid-19 pandemic how scientific journals (especially medical journals) play an important role in keeping the scientific community, health professionals, journalists, managers and other social actors informed and up to date. This unknown disease spread rapidly across five continents and placed science at the center of public debate.

The pace of scientific publications accelerated so that scientific information could be accessed as quickly as possible, including scientific manuscripts that have not yet been subjected to peer review (known as preprints), which have become more widely accessed by journalists (Oliveira et al., 2021; Fleerackers et al., 2022). Although preprints are intended for communication between experts, they are available online quickly and for free, they can be cited, and they were an important source of information during the pandemic. Just two of the world’s most important repositories, bioRxiv and medRxiv, published roughly 33 preprints each day on covid-19 alone in 2020.^1^ In PubMed, one of the main databases of scientific articles in the field of international medicine, there were 64,546 articles on the disease in 2020, or 247 per day. Of these articles, the journals *The Lancet* and the *New England Journal of Medicine* (*NEJM*) published 884 (1.4%) on this topic during the same period.

This scenario gave rise to the “infodemic” (WHO, 2020), a term describing a large volume of information that quickly generates misinformation and rumors via social networks (Zarocostas, 2020), and which placed human lives in jeopardy. During this same period there was also an increase in scientific production (Else, 2020), especially in the area of health and medicine, which grew by around 92%.

Much of the information about the novel coronavirus that helped define social behavior and public health policies came from information made available in preprints or published in editorials, articles, reviews and even bulletins in scientific journals. But access to this scientific information is not enough to impact society, and communication strategies are consequently needed to ensure that non-specialists can access, understand, process and use it.

Aware of their responsibility to make scientific medical information accessible, the scientific journals *NEJM* and *The Lancet* have historically invested in communication strategies, making it easier for their content to reach the public through the mainstream media or directly arrive to scientific and non-specialized audiences via their websites and social networks.

Demoor (2023) states that the fight against disinformation is not new to *The Lancet*, which since its founding has acted as an instrument for exposing misinformation, debate, and proposals to improve public health policies and medicine itself. Even so, the visibility and prestige of both publications does not prevent articles from being retracted or involved in the spread of misinformation, which occurred during the pandemic (Ledford, Noorden, 2020; Prasad, 2021), a period which saw higher numbers of retractions worldwide (Yeo-Teh, Tang, 2021) due to the increased volume of publications and the speed at which studies were approved.

Under normal conditions, responsibility for improper coverage of the medical field usually falls to journalists (Dentzer, 2009). The article by Andrew Wakefield linking the measles-mumps-rubella vaccine to the emergence of autism is one example of the fragile nature of the peer review system, and of journals’ responsibility to control the quality of scientific information. This study was published in *The Lancet* in 1998, and was first questioned by a journalist in 2003 (Noordeen, Hettiarachchi, 2020); it was only retracted seven years after it was found to be fraudulent.

Despite examples of scientific malpractice and improper science communication, medical journals have developed strategies to communicate the information they publish. Within this context, this article analyzes the historical trajectory of these two medical journals, with a focus on communication activities and strategies that extend beyond the academic community.

## From a vehicle for peer communication to reaching society at large

The relationship between science communication and journal visibility is a subject of growing interest in research on information science, especially given the availability of access statistics and sharing of scientific articles on social networks. Some studies have sought out elements to verify that good scientific dissemination, especially on social networks, has a direct and positive impact on journal visibility (Packer, Sales, Rodrigues, 2016).

Barata and Menezes (2013) determined that 28% of science news articles in the *Folha de S.Paulo* newspaper between 2007 and 2011 cited scientific journals as a source of information. Of the 278 journals mentioned, six dominated the news, in a kind of oligopoly: *Nature* or the *Nature* group; *Science* or the *Science* group; *Proceedings of the National Academy of Science (PNAS*); *NEJM*; *Plos ONE*; and the *Journal of the American Association of Medicine (JAMA*), all published in English (the first group in England and the remainder in the United States). This data is corroborated by a study showing that the scientific journals that have received the most media and social media attention include *Science*, *Nature*, *JAMA*, *NEJM* and *The Lancet* (Lehmkuhl, Promies, 2020). These journals also received the most citations in the Web of Science (WoS) index.

Part of the reason that only a few international scientific journals dominate the news is related to the prestige of the studies published, but it also has to do with the ease of access to published scientific information. These journals invest heavily in communication strategies that make it easier for journalists to access their content and work by the authors of the studies that will be published (Packer, Sales, Rodrigues, 2016).

Luiz (2007) analyzed 154 articles in the *Folha de S.Paulo* and *O Estado de S.Paulo* newspapers that cited articles from the journals *NEJM*, *The British Medical Journal (BMJ*), *JAMA* and *The Lancet*, and found that they dedicated little attention to the risks of the research. Another study investigated news coverage of articles in *The Lancet* and *BMJ* in two British newspapers and showed that all the articles had press releases as their source, reproduced their content, and rarely reported on randomized studies with more scientific evidence of effectiveness (Bartlett, Sterne, Egger, 2002). No study attributed the responsibility for directing news coverage to the scientific journals.

One example of working together with the media can be seen in the internationally prestigious journals with over a century of tradition such as *Nature* (from 1869) and *Science* (from 1880). The former emerged with the purpose of “being a vehicle for disseminating science to a wider audience” (Barata, 2010, p.114),^2^ while the latter was intended to be a weekly magazine about advances in science. Since their foundation, journalistic and marketing strategies have made both publications references among the scientific community and the non-specialist public. Both journals have been considered by Barata as hybrid publications, with characteristics of scientific journals as well as magazines disseminating science.

Internationally prestigious journals like these lead discussions on priorities in the world of science, and are consequently responsible for legitimizing science and helping to base the activities of the scientific community on what are known as the dominant paradigms (Kuhn, 1997). These are the scientific journals that set standards and inspire trends to other publications, such as the peer review model, formatting, strict criteria for article approval, media presence, communication with the public, and other aspects of scientific publishing. The credibility of these international journals in the eyes of the worldwide academic community reinforces the dominance of developed countries in global scientific production.

For example, data from the report entitled *Analysis of bibliometric indicators for European policies 2000-2013* (European Commission, 2015), which used bibliometric indicators to measure the quantity and impact of scientific publications in various parts of the world, concluded that the United States published 23% of the articles indexed in the Scopus database during the study period (roughly 19,500 titles from more than 5,000 international publishers, including coverage of 16,500 peer-reviewed journals in the scientific and technical fields and in the medical and social sciences), followed by the UK with 5% of the indexed articles. Both nations remained at the top of world scientific production until 2017, when China took the lead (White, 2019). With the increase in citations of articles in Chinese scientific journals, a factor considered to indicate the quality of these publications, competition for visibility and prestige is also expected to increase.

The practice of science communication has also been an opportunity for scientific journals to expand their mission to communicate with not only specialists (the stated purpose of journals, since their origin in 1665), but also interested parties from other areas of knowledge as well as lay readers. In Brazil, studies have analyzed scientific journals from the viewpoint of altmetrics,^3^ namely how these publications serve to disseminate content to society (Araújo, 2015; Gouveia, 2016). The strategies used by Brazilian scientific publications and the potential for communication outside the walls of academia have proved advantageous in attracting citations, visibility and new audiences, even when resources and staff are limited.

This article analyzes the communication strategies of two international medical journals with more than a century of history, the *NEJM* (lauched in the United States in 1812) and *The Lancet* (launched in the UK in 1823). Both publications enjoy significant prestige in the international scientific community, and lead the rankings that determine scientific impact among medical journals, such as the Impact Factor (IF).^4^ These journals have attained increasing IFs since 2000, and reached extremely high levels in the post-pandemic period. *The Lancet*, for example, went from an IF of 10.232 in 2000 to 168.9 in 2022, while the *NEJM* rose from 29.512 in 2000 to 158.6 in 2022.^5^ Although these journals have mostly restricted access (for subscribers only), they are increasingly concentrating citations and, in turn, prestige in the worldwide medical community. Part of this prestige seems to be reinforced by ongoing efforts to maintain visibility through communication strategies (Grady, Wetterlow, 1978).

Our analysis focuses on the role of communication to non-specialized audiences in the medical journals *NEJM* and *The Lancet*, based on the historical trajectories of both journals in order to identify how communication has been incorporated into their policies.

This effort involved the following research questions:

1. Historically, what role has communication of medicine to the non-expert public played in two of the world’s most prestigious medical journals, *The Lancet* and *NEJM*?
2. Has dissemination to non-specialized audiences gained relevance throughout the history of medical journals?

To answer these research questions, we will analyze the editorial trajectories and communication strategies of the *NEJM* and *The Lancet*.

## Methodology

The corpus used in this article is composed of 47 publications from both medical journals, including “commemorative” articles marking significant anniversaries, on editorial changes related to communication strategies: 19 from *NEJM* and 28 from *The Lancet* (for supplementary material, see Barata, Medeiros, 2025).

In this study, the journals’ own search tools were used to locate commemorative editorials or those related to communication and science communication. The search for keywords in the WoS^6^ proved relatively unsuccessful, since the search does not differentiate editorials from articles or other formats, and we opted to search the archives of both journals. The search for commemorative editorials was carried out every five years, on the launch date of each journal. The keywords used were: anniversary OR celebration OR celebrate AND communication OR journalism.

A supplementary search was carried out for other publications (commentaries, articles, reviews and news) mentioned in the first sample, as well as publications from other journals. The content was then analyzed to identify the main editorial changes, investments in staff, and communication strategies adopted.

### The New England Journal of Medicine (NEJM)

The first issue of the *New England Journal of Medicine and Surgery and the Collateral Branches of Science* was published on January 1, 1812, in Boston in the United States (Podolsky, Greene, Jones, 2012, p.1457). It appeared 15 years after the emergence of the first American journal, the *Medical Repository*, in New York in 1797 (Green, 1923), and began as a quarterly journal, which in 1828 was incorporated into the *Medical Intelligencer* and renamed *Boston Medical and Surgical Journal* (Garland, 1928), and became a weekly publication. It was sold again in 1921 and came under the responsibility of the Massachusetts Medical Society, where it received funding that ensured its financial sustainability and, in 1927, it was renamed *New England Journal of Medicine* (Garland, 1928).

The original objective of *NEJM* was to speak to medical students, since no journal in the country at that time fulfilled this mission; today it is considered one of the most prestigious medical journals internationally, as indicated by the high number of citations (its IF in 2022 was 158.5) and the social attention its articles receive (it is always among the top five or ten scientific journals in terms of highest social attention score, according to the Altmetric Attention Score).

In a commemorative editorial, historian of medicine at Harvard University Allan M. Brandt (2012) mentioned that during its 200 years of circulation, the journal not only evolved structurally but has always been concerned with publishing major medical discoveries and studies related to American medical education, especially in the United States (Campion et al., 2010).

From its creation, NEJM has remained firm in its aim of creating dialog with medical professionals (Fishbein, 1928).

The NEJM followed a tradition of medical journals started by the French journal *Nouvelles Découvert sur Toutes les Parties de la Médecine* (launched in 1679 and closed in 1681), followed by the journal *Medicina Curiosa*, the first English publication in 1684 (Fishbein, 1928), the *Medical Repository*, launched in 1797, the *Philadelphia Medical Museum*, created in 1804 as the first American medical journal launched in New York, as well as other publications in the Boston area, which was industrialized and home to medical schools. Fishbein also gives the credit for the first weekly medical publication to the *Medical Intelligencer*, in 1823, also in Boston, the same year that *The Lancet* was created in London, also with weekly publications.

In 1810, the northeastern United States already had six medical schools with roughly 1,375 students, a figure that would reach 4,338 10 years later (Fishbein, 1928). These medical courses responded to a demand from a populous region with a large influx of migrants that fostered epidemic outbreaks of tuberculosis and smallpox (even though a vaccine already existed), as well as pressure for a genuinely American education that was less dependent on England. Within this context, medical journals offered a means of circulating information quickly, with national and international updates for professionals and students, and were consequently a way to strengthen the national medical community and medicine. Half of the medical population was poorly trained (Fishbein, 1928), so journals also advanced the education of non-academic doctors and new standards of writing and scientific rigor. Fishbein called medical journalism what the editors of weekly medical journals practiced in *JAMA* and the *Boston Medical and Surgical Journal* (or *NEJM*).

In terms of structure, the journal’s first major change occurred in 1828, when it (experiencing financial difficulties) was incorporated into the *Medical Intelligencer* to form the weekly *Boston Medical and Surgical Journal* (Rothstein, 1987). The editorial stated that

during its first hundred years, the journal stood out as the oldest representative of medical journalism in New England and a possible exception in America, with an auspicious future that was expected to continue to embody the character and scientific progress of the profession in New England (Garland, 1928, p.1).

The 50th anniversary of the journal was counted starting from its merger on February 19, 1828, a period when there were only eight medical journals in the United States (Billings, 1879). Although there were 54 medical journals in the country in 1879, only two others were as old as or older than *NEJM*, both from Philadelphia. This surprising fact indicates enormous productivity, compared to a total of 372 medical journals worldwide. The Billings editorial makes it clear that the term “journalism” was used to describe the editor’s practice of medical writing, with the freedom to express his opinion and criticism.

In 1873, the journal came to be published weekly, with a maximum of five articles, rising to 12 (Kassirer, 1999). At the end of the nineteenth century, the number of scientific articles per issue expanded to 15, followed by a change in assessment of articles, which from this time began to be conducted by more than one specialist, giving rise to peer review (Fishbein, 1928).

In the year commemorating the journal’s first 100 years, when it was still called the *Boston Medical and Surgical Journal*, an editorial expressed concern about advertisements that mimicked medical news in newspapers and often misled the uninformed public about treatments that were medically unproven or could be potentially harmful (Garland, 1928). This conflict between publicity and autonomy in publication returned to the *NEJM* in the 1950s.

In 1999, the firing of *JAMA*’s editor-in-chief reignited the debate on the importance of scientific journal editors and the media having autonomy from the decisions and opinions of the journal owners or medical associations (Campion et al., 2010). His firing not only led to criticism from editors of other medical journals (like *NEJM*), but also gave visibility to the influence of medical journals like *JAMA* on public policy.

In 1921, the *NEJM* became part of the Massachusetts Medical Society, a trend followed by countless other journals in pursuit of support from scientific societies, to ensure financial stability on the one hand and visibility on the other. The support and resources that this society began to invest in the journal included maintaining (among other factors) that society may properly endeavor in the broadest manner to bring the achievements of medicine to the attention of all practicing physicians and to disseminate the knowledge of hospital facilities for the instruction of students, as well as the treatment of the sick, and meeting the mission of the institution, which says that health is essentially necessary for the happiness of society (Brandt, 2012).

During the twentieth century, competition grew not only between medical journals, but also between these periodicals and journalism. Concerns with safeguarding medical information led editor Franz J. Ingelfinger to establish one of the most significant changes in medical coverage. In 1969, the “Ingelfinger rule” established the journal’s right to publish only new articles that had not been published elsewhere, including in the press (NEJM, 1969; Relman, 1981). This rule came about after a series of criticisms received by the editor at that time, after refusing to publish a report that had been scheduled to appear in the journal but was published earlier by the *Medical World News* (Relman, 1981). “Critics say that this rule restricts the free flow of information, whereas proponents claim that the information from a paper released early may be inaccurate because the paper has not been subjected to peer review” (Altman, 1996, p.1382).

The rule, which was later adopted by other scientific journals, was not only a way for the journal to have exclusivity over the content it published, but Ingelfinger also believed that doctors were best-equipped to assess the quality of medical articles, and that premature disclosure of medical research and still-undocumented studies could cause confusion among laypeople. “We believe strongly in the importance of an informed public and the public’s right to know, but we also believe that it is important to take time to make sure that the public is not misled” (Relman, 1981, p.825).

Faced with this concern, the journal recognized and legitimized the need for and importance of the media’s work, since, in the view of its editors, “the popular press can play a very important role in reporting and explaining new developments to the public” (Relman, 1981, p.825).

Despite defending the journalistic work of the mainstream media, the *NEJM* considered itself capable of publishing not only scientific evidence but also news about recent scientific discoveries, not restricting itself to technical texts. In this way, it broadened its readership (Grady, Wetterlow, 1978) and also guaranteed the exclusivity and survival of this medical journal in a competitive market.

In 1977, *NEJM* became the first scientific journal to receive the George Polk Award for Journalistic Excellence in the Science Reporting category (NEJM, 1978; Campion et al., 2010), indicating its continued relevance in disseminating medicine to a non-specialized audience.

Ten years later, in celebrating 175 years in press, an editorial by Arnold S. Relman (1987) shared technical innovations that improved the color quality in illustrations and the value of its publications. He also called attention to the importance of sharing its content with the press, which received the studies in advance so that they could be published on Thursdays, along with the new issue of *NEJM* (Kassirer, 1999). The journal’s communication gained momentum and visibility with the development of the internet and social networks, spaces where it continues to operate intensively today.

### NEJM in the digital environment

The journal invests heavily in social networks, with agile and varied content that results in significant interaction, which can be seen on both the website and the social networks. In this way, *NEJM* has established itself as one of the most important scientific journals in the field of medicine, reinforcing its mission: to address issues of social interest, without neglecting dialog with professionals in the field. The results can be seen in the journal’s significant results related to IF, altmetrics and followers on social media. This intense work in the online environment began in 1996 with the launch of the journal’s first website, which only published the abstracts of the articles in the print version. They were posted manually, on a weekly basis (Lancet, 2012).

The *NEJM* website now publishes articles seven to nine weeks ahead of the print edition. This practice was intended to ensure that articles that could impact medical practice were available quickly (Brandt, 2012). “Our aim is to continue to develop new options for electronic information delivery that will be of interest, use, and value to all” (Campion et al., 2010, p.1175).

In the early 2000s, rather than reproducing the articles in the print version, the website began to use a new format to communicate with readers: video, a medium that the journal’s editors deemed highly effective for communicating complex biomedical information in a way that the press cannot since it is limited to text and still images (Brandt, 2012).

In 2007, the articles, images and explanatory videos gained a new space: the social media outlets Facebook and Twitter (now X). In November 2024, *NEJM*’s X page had more than 945,000 followers on its main profile, while its Facebook page had over 1.8 million followers, along with 553,000 on Instagram.

According to data from the journal’s own website, every week roughly one million readers receive the journal summary via email (through news agency mailings), and every month around 2.5 million users log on to the website. The digital edition of the journal has also permitted restricted access (some articles are open access) for users from over a hundred countries (Campion et al., 2010).

In 2012*,* following a trend at other scientific journals (*Cell*, *Nature*, *Science* and *The Lancet*), the *NEJM* Group was created. This group incorporated the flagship journal, along with *NEJM Journal Watch*, considered the “independent voice within the group” *(* Lancet, 2024), as a blog independent from the journal’s website where medical authors and editors curate and summarize research from over 250 medical journals as well as other sources of medical news. Later, *NEJM Catalyst* was created with the objective to “bring together health care executives, clinical leaders, and clinicians to share innovative ideas and practical applications for enhancing the value of healthcare delivery” (Lancet, 2024).

This was followed by the creation of *NEJM Knowledge+*, a section that gathers questions from readers that are answered by over three hundred authors and editors, and the *NEJM Career Center,* a set of job search tools for physicians as well as articles on medical careers and employment trends. There is also the “Image Challenge” section, which features a weekly challenge containing a photo of a patient with a description of symptoms; it is intended for public interaction, but clearly appeals to medical students who can later check their guesses against the correct diagnosis. The journal’s editorial team is divided into eight working groups: editors, editorial board, editorial systems (which work on *NEJM* Group journals), online editorial group, manuscript editing, graphic arts, editorial production technology (editing all content in an attractive format), and the perspective group (which verifies that the content is within scope) (Müller, Duff, Stern, 2012).

On the occasion of the journal’s bicentennial, Kohane, Drazen and Campion (2012, p.2539) predicted that in the next 100 years, “societies will come to accept that comprehensive knowledge of disease, prevention, and effective treatment is an essential public good,” and it took less than a decade for this statement to become so crucial amid the covid-19 pandemic. They added that “the increasing power of information and communication technologies can help find ways to improve global health” (p.2539).

Also in celebration of 200 years of publication, the journal reinforced its mission: “Our focus remains on the entire medical community, with its scientific knowledge and advances in clinical care” (NEJM, 2012, p.383). But in 2019, with the change of editor-in-chief, the journal reaffirmed its commitment: “After all, our mission is not really to publish a journal. It is, instead, to communicate the most important medical information to providers and their patients” (Rubin, 2019, p.1069). And such a varied audience requires publications with different styles and formats to make the content easier to understand and more readable for those looking to keep up to date with their professional area, medicine and health in general.

### The Lancet

In the nineteenth century, the British Empire experienced a period of intense cultural and artistic production. On the other hand, professional medicine was still in its infancy and remained unregulated, as were medications, and doctors struggled with diseases they knew little about and which had ineffective treatments. This era was also marked by the precariousness of surgery, the absence of anesthesia and the lack of hygiene.

It was against this backdrop of chaos in the medical field that the British surgeon Thomas Wakley (1795-1862) founded the medical journal *The Lancet* on October 5, 1823, with the aim of addressing in its pages what was to be the greatest political objective: to reform the medical system in the United Kingdom *(* Lancet, 1923). Advised and influenced by his friend, the radical political journalist William Cobbett, Wakley saw writing as the best weapon to attack medical misconduct (Jones, 2009; Lancet, 1923). “Wakley had become increasingly aware of widespread corruption within the medical profession, where influence counted for more than ability and where ignorance and avarice were rampant” (Jones, 2009, p.406).

The first editorial already promised to “convey to the public, and to distant practitioners as well as to students in medicine and surgery, reports of the Metropolitan Hospital Lectures” (Lancet, 1823, p.1). On the centennial, the editorial reinforced that, from the outset, “the content was nominally directed toward both the public and the medical reader, [for whom] familiarity with professional situations was presupposed” (Lancet, 1923, p.698).

In 1832, Wakley ran for Parliament for the first time and lost; he tried again in 1835, won and remained in office for 17 years. He was among those responsible for the content of the 1858 Medical Act, which established the General Council of Medical Education and Registration (Jones, 2009, p.405). Throughout this period, he fought for the reform of forensic medicine, public health and other medical issues, and campaigned to reinvent the coroner’s office as an open, democratic and medically competent institution. Another cause Wakley defended was English sanitary reform, as seen in an 1862 editorial in which he describes the health problems faced by the English as a result of the lack of basic sanitation, a very different reality from the situation at Windsor Castle at the time (Jones, 2009).

Since the first issue of *The Lancet* in 1823, the journal strove to disseminate reliable, current and relevant medical information to medical researchers and practitioners, medical students and individuals in general. The lectures given by Astley Paston Cooper and other acclaimed professionals became a hallmark (Lancet, 1823). Wakley was troublesome because of his critical stance, often denouncing medical institutions, public health policies, medical schools and issues related to the medical community itself, which led to numerous libel suits during the first decade of the journal’s existence (Garner, 1973). The work of Wakley and *The Lancet* contributed to improvements in early nineteenth century medicine (Garner, 1973), scientific publishing and medical journalism (Jones, 2009).

According to Conrad Keating (2023), a doctor at the School of Medicine at Trinity College Dublin, many of the discoveries in the medical field, such as clinical trials and epidemiological studies (which analyze the determinants of health and disease among individuals and society) were first reported in this journal. Other authors reinforce its mission not only in technical-scientific terms, but also in terms of medical policies.

From the first days of *The Lancet* 200 years ago, surgery has had a central role in its existence. The journal has acted as a nerve centre, linking surgical innovation by individuals with challenge and debate in the wider profession (Frampton, Kneebone, 2023, p.1222).

Another opinion piece highlighting the English journal’s “relationship” with society was emphasized in “*The Lancet* and the medical journal as an instrument of reform” (Podolsky, 2023, p.1220), which addressed its identity: an instrument of reform in medicine and society and, crucially, for redefining the boundaries between the two.

But it was the editorial “*The Lancet* at 200 years: timeless mission to drive positive social change through advancing medical research and science” (Lancet, 2023, p.1200) that emphasized the journal’s social mission:

*The Lancet* was founded in 1823 by Thomas Wakley with the vision to drive positive social change by advancing medical research and science for the greater good, by addressing inequities of the time with access to medical knowledge for “medical and surgical practitioners” in the UK and the British colonies (as they were referred to at the time), and by being more than a medical journal.

Wakley remained editor of the journal until his death in 1862, when his son Thomas Henry Wakley took over the role. After graduating in medicine, he went on to fight the battle of the medical student (Lancet, 1923, p.700). The younger Wakley used the pages of the journal to denounce situations where the law was being distorted, but did not cease to talk to physicians. This interest in promoting changes in the English health system, as well as in the country’s public health, was evident in the pages of issues from the nineteenth century, such as on October 1, 1831, when the journal dedicated an issue to the cholera epidemic that was ravaging Europe at the time, and a special issue on alternative treatments for patients with dementia (Lancet, 1923).

It is worth noting that a key period in the history of *The Lancet* was reached in 1850, when the journal had been established for more than a quarter century and enjoyed a reputation for solidity, frankness and major effort, which spread across the country and the continent (Lancet, 1923).

In 1973, when celebrating 150 years, the *Canadian Medical Association Journal* dedicated the editorial of its 150th issue to its opinion on the role played by *The Lancet* since its creation. And in 1972, the US Institute for Scientific Information tabulated the fifty most cited scientific journals in the world: and *The Lancet* ranked 11th.

The 1923 issues of *The Lancet*, as a way of celebrating the hundred-year anniversary, highlighted in their editorials the topics that aroused the greatest interest among readers. These included the first experiments with nutrition – the anatomical activities, sophistication of food, medical conduct and the reform of the English health system brought about by the Dawson Report^7^ (Lancet, 1923, p.700). This report established guidelines that significantly influenced the British national system in 1948, which went on to guide the reorganization of health systems around the world (Lancet, 1923).

On this subject, the editorial “Medicine and the public” (Lancet, 1923) recalls Wakley’s first attempt to enter the English Parliament and the significant change seen in the journal’s pages as a result: character. There was no effort to disseminate general information, but rather an effort to show the universality of medicine itself, permeating all of life’s circumstances and activities. It can be said that throughout the nineteenth century, the journal constructed an image of connections and pioneering spirit by creating dialog with physicians and medical students and portraying a society that yearned for changes in its health system.

With the turn of the century and the change in the English health system, articles on pioneering studies involving diseases that plagued humanity in various countries began to feature prominently in the pages of *The Lancet*; these included malaria and yellow fever. It was also at this time that clinical pathology and public health began to gain ground in the journal (Lancet, 1910).

Peer review was debated in its pages in 1993, in the article “Early announcements,” where the journal argued that early disclosure of the results of a clinical trial (prior to peer review) could lead to distorted information. This view was also defended by the International Committee of Medical Journal Editors, of which the English journal was a member (Lancet, 1993).

The relationship between science and the media has been the subject of other editorials and articles throughout the journal’s history; one notable example is the article by Fiona Fox (2014), in which she discusses the reasons why scientists should involve the media in their research. For this author, most scientists are publicly funded, so the public has a right to know how this money is being spent and the directions in which scientists are progressing, an argument that is still used today. She also points out that it is a good opportunity to put science into the public domain at the very moment when the general public really cares.

There is also the text “A health scare in the mass media,” where the journal discusses the issue of embargoes on publishing about new studies as well as the journalist’s prior knowledge in specific areas of science (such as epidemiology, for example), in an attempt to avoid the misreporting of health discoveries in the media, an issue that worried scientists and doctors (Lancet, 2002).

The topics of the articles have not only changed, however, but the volume of articles increased with each issue. The issues gained editorials, which in turn led to the arrangement of articles into categories: editorials, clinical studies, public health, and comments and news. The twenty-first century was also marked by other impactful articles which, as in the previous century, portrayed the society at the time, granting *The Lancet* status as “one of the leading medical journals of our time” (Jones, 2009, p.404).

In October 2010, evidence was gathered on how the mass media could influence health, reinforcing the concern that the journal has always shown about communicating with peers and non-specialists. The debate in question was about the exposure of young people to unhealthy behavior through different communication channels.

In early 2022, one year short of 200 years of uninterrupted publication, *The Lancet* published an editorial entitled “The state of science and society in 2022,” a debate on how science has often been attacked. In the midst of the covid-19 pandemic, the journal gathered facts, reports and research findings that showed how anti-science rhetoric has gained strength around the world, causing scientists to suffer various kinds of threats, and how damaging this discourse was in many countries in terms of how the pandemic was addressed, especially in Brazil (Lancet, 2022).

An analysis of the archive shows that over nearly 200 years, *The Lancet* has kept pace with social and technological changes in society as a whole. One example was the change in the journal’s structure, with regard to the peer review process. This model still exists today, but it shares space with the fast track model. This new scheme, which is widely used by health publications, accelerates the article review stage (and, occasionally, other processes along with it), hastening publication time *(* Lancet, 2015).

Given the quality criteria of international indexers for online academic publications, the entire process of evaluating articles by journals ideally should not exceed 90 days. In fast track mode, this can drop to 28 days or less, and peer review can be done within 72 hours.^8^ The main justification for journals adopting this accelerated evaluation method is to provide rapid visibility to articles that make a major contribution to their field and have the potential for immediate practical implications.

### The Lancet in the digital environment

An important milestone in the journal’s structure came in the early 1990s with the advent of the internet, when *The Lancet* created its first website in addition to the print editions. On the site it provided scanned editions from the previous 100 years, and also abstracts of the articles in the print version of the journal. The content was posted manually, on a weekly basis (Lancet, 2012).

According to its current website, *The Lancet* has its own social media team, which the journal calls the “Online editorial group,” made up of professionals who are experts in electronic content and web production (Lancet, 2012). It was only in 2010, when the site was redesigned and updated to its current format, that the entirety of the journal’s collection was offered there (for a fee). Since then, readers have access to the journal’s digitized collection and all the “*Lancet* family” journals, as well as articles from the current issue of the main journal and a wealth of promotional material, as described on the journal’s website.

While the focus of the journal is to be an important channel for medical information and to keep up with new trends in society, inclusion of the social networks is also part of the spectrum of changes that *The Lancet* has undergone during the twentieth century. Although social networks were already being considered in 2008, it was only in 2010 that the journal consolidated its presence on Facebook, and on Twitter (now X) in 2021, which made the interactive content produced by its team more readily available.

In November 2024, *The Lancet*’s X page proved most popular, with 749,000 followers, while on its Facebook page (which highlights the slogan “More than a medical journal”) there were 356,000 followers, and a smaller presence on Instagram with just over 18,000 followers. These channels use written and audiovisual material to address the different topics published in the journals of the “*Lancet* family” in a simple and objective manner.

The journal’s team is composed of editors, ombudsmen, and reviewers, and the publication also has a specialized communications department, professionals who specialize in producing content for social networks, news agencies and journalists.

In 2015, another significant change took effect, the insertion of “Research in Context,” a section that, along with other items, became indispensable when submitting an article to the journal. This section, as defined by the journal itself on its website, “should include a description of all the evidence that the authors considered before undertaking this study” (Lancet, 2015).

The journal’s page on X combines the work of disseminating new articles, issues and potential topics for changes in the field of health policy. Its target audience is professionals in the medical field, since it is largely concerned with disseminating and promoting public policies and bringing public health issues to a less specialized audience. Altmetric, a company that measures the performance of articles on digital platforms, indicated that X has been playing an important role outside university walls; this may indicate investment by *The Lancet* and *NEJM* in this social network which, despite having fewer users than other social networks, concentrates followers who may sway public opinion, such as politicians, journalists, scientists and influencers considered strategic.

Patients use social media to research their symptoms, their doctors, their treatments, and to set up support and information groups. Clinicians can use social media to drive awareness, to provide accurate information, and as a portal to communicate with other physicians (Lancet, 2012, p.1562).

## Discussion

This article brings together the results of an analysis of the history of the scientific journals *NEJM* and *The Lancet*, in order to understand how these journals relate to communication with society as a whole, while also pointing out the main practices of these journals in disseminating their articles and the impact generated through these communication practices.

This process was made possible by bibliographic review of scientific articles and particularly editorials by the journals themselves, which makes it possible to affirm that communication beyond specialists in the field has always been part of the universe of these two publications. One example worth highlighting, in the case of *NEJM*, is the statement by then-editor Arnold S. Relman, in an editorial dated October 1, 1981, where he reinforced that the role of newspapers is different from that of scientific journals, and that newspapers should only publish the news of science at the same time or after it has been published by specialized journals. He emphasized his concern about the quality of media coverage of scientific articles at the time; for him, many details were left out (Relman, 1981, p.824).

A very important aspect of *The Lancet* is that in the very first editorial, the editor at that time, Thomas Wakley, explained the role that the journal intended to play (and which it still plays today): to use scientific articles to promote debate on issues related to health policies in the United Kingdom, to publicize major scientific discoveries, and to promote dialogue with medical students. The journal expanded its activities and became an important voice and promoter of public health policies around the world. One example of this is *The Lancet* Countdown initiative, organized by *The Lancet* and the Wellcome Trust, since 2016, to ensure implementation of public health policies in light of the impacts caused by climate change in various regions of the planet.

Although the two scientific journals have had different target audiences since their launches, both have been concerned with seeking out and ensuring dialog with audiences other than scientists. Before the advent of the internet, as we have seen throughout this analysis, this work was done through scientific events and newsletters. With the arrival of the internet, and especially social media, this work has gained even more strength and significance within the two scientific journals.

During the first years of publication, talking about science in language accessible to all audiences was something that took place through the journals’ own editorials. They objectively presented the main information from the articles that headlined each issue; in other words, they did not just present what the reader would find in the edition, but offered explanations in accessible language on topics that were often complex for non-medical professionals. Another way publications found to make the content of their new editions accessible was through dialog with journalists.

However, both *NEJM* and *The Lancet* expressed reservations about journalistic practice, which led to efforts to disseminate their own content in an attempt to minimize misinformation about the research published on their pages. As a result, these journals began to produce material and send it to journalists to be distributed into society. Since the advent of the internet, this work has been done through press releases from news agencies, but also directly through the communication channels of the journals themselves.

Indicators of social attention toward science, measured through data from Altmetric.com, show that both *The Lancet* and *NEJM* had major visibility on digital platforms among the hundred scientific articles published by the international media between 2013 and 2020, bringing the findings of their studies out beyond the academic public. This is an effort by these and other scientific journals to invest in the public communication of science with greater visibility, along with attention to public interest in the subject of health and medicine, which ultimately are strongly related to the scientific credibility gained from this exposure.

## Final considerations

The editorial trajectories and dissemination strategies of the *NEJM* and *The Lancet* show that communication has always been present in these journals’ concerns to reach the public beyond scientists, and as a fundamental tool in their daily editorial activities. With the presence of social networks and direct communication with society without moderation by journalists, these journals are increasingly using strategies to expand the reach of their audience, made up of managers, journalists, health professionals, patients and health students (in other words, a strategic audience), so that they receive relevant medical information.

For the scientific editors of these journals, we can be sure that the ways to increase visibility, expand their reach to diverse audiences (with a focus on medical students and health professionals, in the case of *NEJM*), and influence changes in public health policies (more strongly in the case of *The Lancet*) lie in science communication as an efficient and relevant strategy in the editorial activities of both journals, with greater engagement in social networks and efforts to communicate with non-specialists by *NEJM*.

Finally, it is worth noting that there is professionalization and investment in marketing, publishing and communication strategies which have allowed these journals to remain references in the field of medicine, both in academia and in the media. They have both garnered enormous public and journalistic interest, as a result of their visibility on social networks and their communication efforts.

The engagement and investment by *The Lancet* and *NEJM* serve as models for journals in medicine and other knowledge areas to rethink their mission in science communication, with the possibility of reaching audiences beyond the experts in the specific areas in which they operate, their social responsibility, and their visibility and prestige in academia and society. Despite efforts to communicate and control the editorial flow of articles, these journals continue to face the challenge of guaranteeing that the content they publish is credible, high quality and socially responsible.

This study was limited to a small sample of publications containing the journals’ missions, editorial policies, changes, and criticisms of their relationship with the public and society, based on the anniversary celebrations of *The Lancet* and *NEJM*, and by no means exhausted the subject. Further studies could be carried out using quantitative or content analysis to examine other aspects of public communication in medicine.

## Data Availability

https://doi.org/10.25824/redu/BKS5UN.

## NOTES

1 On February 11, 2021, bioRxiv and medRxiv allowed access to 11,918 preprints on covid-19, which were made available in 2020.

2 In this and other citations of text from Portuguese, a free translation has been provided.

3 Altmetrics, or complementary metrics, measures and quantifies the social attention an article receives on digital platforms like blogs, Wikipedia, news articles, Facebook or X posts.

4 The IF has been used to measure the impact, prestige or quality of a scientific journal, despite numerous criticisms of its use.

5 According to data from the *Journal Citation Reports*, shared on the websites of each journal.

6 Web of Science (WoS) was chosen because it was the international literature indexer with the most structured search fields for research in 2018, when this research began. There are currently other databases, such as Dimensions (2018) and OpenAlex (2022), which could be used for new analyses in the future.

7 This report was drafted by the British Ministry of Health in 1920 and is considered one of the first documents to use the concept of primary health care from the perspective of a regionalized and hierarchical systemic organization of health services, by level of complexity and on a defined geographical basis.

8 Information originally taken from *The Lancet*’s *website* in July 2015, but updated in August 2025. “Information for authors.” Available at: https://www.thelancet.com/lancet/about. Accessed: 15 Aug. 2025.
