# Supplementary material for: The scientific journal, beyond experts: the communication strategies of The New England Journal of Medicine and The Lancet
Source: Hist Cienc Saude Manguinhos. 2026 May 11;33:e2026005. doi: 10.1590/S0104-59702026000100005en (PMC13170114; doi:10.1590/S0104-59702026000100005en)
Supplement: Translation

# Translation: A revista científica para além de especialistas: as estratégias de comunicação dos periódicos The New England Journal of Medicine e The Lancet

**DOI:** 10.1590/S0104-59702026000100005

**Authors:** Germana Barata, Carolina Ferreira Medeiros

**Affiliations:** i Pesquisadora, Laboratório de Estudos Avançados em Jornalismo/Núcleo de Desenvolvimento da Criatividade/ Universida de Estadual de Campinas. Campinas – SP – Brasil. germana@unicamp.br; ii Pesquisadora, Instituto de Biologia/Universidade Estadual de Campinas. Campinas – SP – Brasil. carolinafmedeiros07@gmail.com

**Keywords:** Divulgação científica, História da medicina, Revistas médicas, The Lancet, The New England Journal of Medicine

## Abstract

A área da saúde é pioneira no diálogo com a sociedade, a mídia, os profissionais de saúde e tomadores de decisão, em função do grande interesse público e de sua responsabilidade social. Duas revistas médicas bicentenárias de prestígio internacional – *The New England Journal of Medicine* e *The Lancet* – são exemplos de estratégias de comunicação que vão além dos muros da academia. O artigo analisa políticas editoriais, editoriais comemorativos e estratégias de comunicação das duas revistas para entender a relação com o público ao longo da história. A análise mostra que a divulgação para não especialistas é parte importante das políticas editoriais e se ampliou ao longo dos anos em ambas as revistas.

## O papel social cada vez mais relevante das revistas científicas

Em tempos de pandemia da covid-19 ficou evidenciado o importante papel que as revistas científicas, sobretudo as médicas, desempenham para manter a comunidade científica, os profissionais da saúde, os jornalistas, os gestores e os demais atores sociais informados e atualizados. Essa doença desconhecida se espalhou rapidamente pelos cinco continentes e colocou a ciência no centro do debate público.

O ritmo das publicações científicas foi acelerado para que as informações científicas ficassem acessíveis o mais rapidamente possível, incluindo os manuscritos científicos ainda sem revisão por pares, os chamados *preprints*, que passaram a ser mais acessados por jornalistas (Oliveira et al., 2021; Fleerackers et al., 2022). Apesar de voltados para a comunicação entre especialistas, os *preprints* são disponibilizados *on-line* de forma gratuita e rápida, podem ser citados e foram importante fonte de informação durante a pandemia. Dois repositórios dos mais importantes do mundo, o bioRxiv e o medRxiv, publicaram sozinhos cerca de 33 *preprints* por dia apenas sobre covid-19 em 2020.^1^ No PubMed, uma das principais bases de artigos científicos da área médica internacional, havia 64.546 artigos sobre a doença em 2020, ou 247 por dia. Entre esses artigos, as revistas *The Lancet* e *The New England Journal of Medicine* (*NEJM*) publicaram 884 artigos (1,4%) sobre o mesmo tema no período.

Esse cenário deu origem à infodemia (WHO, 2020), termo que descreve um grande volume de informações que gera desinformação, rumores, de forma rápida, via rede social (Zarocostas, 2020), que causou riscos para a vida humana. Nesse período, também houve aumento na produção científica (Else, 2020), com destaque para a área de saúde e medicina, que cresceu cerca de 92%.

Boa parte das informações sobre o novo coronavírus, e que ajudaram a definir o comportamento social e as políticas de saúde pública, veio a partir de informações disponibilizadas em *preprints* ou publicadas em editoriais, artigos, revisões e até notícias de revistas científicas. O acesso a essas informações científicas não é suficiente para impactar a sociedade, portanto, é necessário que estratégias de comunicação garantam que não especialistas acessem, compreendam, processem e as utilizem.

Cientes da responsabilidade de tornar acessíveis as informações científicas médicas, as revistas científicas *NEJM* e *The Lancet* historicamente investem em estratégias de comunicação, facilitam a chegada de seus conteúdos ao público por meio da grande mídia ou diretamente para os públicos científicos e não especializados por meio de *website* e redes sociais.

Demoor (2023) afirma que o combate à desinformação não é novo para *The Lancet*, que tem atuado, desde sua origem, como um instrumento de denúncias, debates e propostas para melhorar as políticas de saúde pública e da própria medicina.

A visibilidade e o prestígio de ambas as publicações não impedem, no entanto, que haja artigos retratados e envolvidos na disseminação de desinformação, como ocorreu durante a pandemia (Ledford, Noorden, 2020; Prasad, 2021), período em que aumentou o número de retratações pelo mundo (Yeo-Teh, Tang, 2021), em função do aumento do volume de publicações e da velocidade de aprovação de estudos.

Normalmente, porém, a responsabilidade sobre a má cobertura da área médica recai sobre os jornalistas (Dentzer, 2009). O caso do artigo de Andrew Wakefield, que associou a vacina da tríplice viral ao surgimento de casos de autismo, é exemplo da fragilidade do sistema de avaliação por pares e da responsabilidade das revistas no controle de qualidade das informações científicas. Publicado em 1998, em *The Lancet*, o estudo foi primeiramente questionado por um jornalista em 2003 (Noordeen, Hettiarachchi, 2020), e, depois de constatada fraude, levou sete anos para ser retratado.

Apesar de exemplos de práticas de má condução científica e de divulgação científica, as revistas médicas desenvolveram estratégias para comunicar a informação que publicam. Nesse contexto, este artigo analisa a trajetória histórica de duas revistas médicas com enfoque nas ações e estratégias de comunicação para além da comunidade acadêmica.

## De veículo de comunicação para pares até chegar à sociedade

A relação entre divulgação científica e visibilidade dos periódicos é tema de crescente interesse nos estudos da ciência da informação, sobretudo a partir da disponibilidade de dados estatísticos de acesso e do compartilhamento de artigos científicos nas redes sociais. Há estudos que buscam reunir elementos para constatar que uma boa divulgação científica, em especial nas redes sociais, impacta de forma direta e positiva a visibilidade de um periódico (Packer, Sales, Rodrigues, 2016).

Barata e Menezes (2013) analisaram que 28% das notícias publicadas na *Folha de S.Paulo*, entre 2007 e 2011, sobre ciência citavam revistas científicas como fonte de informação. Das 278 revistas científicas mencionadas, seis dominaram o noticiário, em uma espécie de oligopólio: *Nature* ou grupo *Nature*; *Science* ou grupo *Science*; *Proceedings of the National Academy of Science* (*PNAS*); *NEJM*; *Plos ONE*; e *The Journal of the American Association of Medicine* (*JAMA*), todas em inglês, a primeira publicada na Inglaterra e as demais nos EUA. Esses dados são corroborados por estudo que mostra que entre as revistas científicas que receberam mais atenção midiática e nas redes sociais estão *Science*, *Nature*, *JAMA*, *NEJM* e *The Lancet* (Lehmkuhl, Promies, 2020). E são também as que receberam mais citações no indexador Web of Science (WoS).

Parte da razão para existir um predomínio de poucas revistas científicas internacionais prevalecendo no noticiário está relacionada ao prestígio dos estudos publicados, mas passa também pela facilidade de acesso à informação científica divulgada. Essas revistas investem pesadamente em estratégias de comunicação que facilitam o acesso de jornalistas aos seus conteúdos e aos autores dos estudos que serão divulgados (Packer, Sales, Rodrigues, 2016).

Luiz (2007) analisou 154 matérias nos jornais *Folha de S.Paulo* e *O Estado de S. Paulo* que citavam artigos das revistas *NEJM*, *The British Medical Journal* (*BMJ*), *JAMA* e *The Lancet* e avaliou que elas pouco tratavam dos riscos das pesquisas. Outro estudo investigou a cobertura jornalística de artigos de *The Lancet* e *BMJ* em dois jornais britânicos e mostrou que todas as notícias tiveram comunicados de imprensa como origem, reproduziam seus conteúdos e raramente divulgaram estudos randomizados, com mais evidências científicas de efetividade (Bartlett, Sterne, Egger, 2002). Nenhum estudo atribui responsabilidade às revistas científicas para direcionar a cobertura jornalística.

Exemplos de empenho junto à mídia são as centenárias revistas internacionais de prestígio internacional como *Nature* (de 1869) e *Science* (de 1880). A primeira surgiu com o propósito de “ser um veículo de divulgação da ciência para um público mais amplo” (Barata, 2010, p.114), enquanto a inglesa nasceu com a proposta de ser uma revista semanal sobre os avanços da ciência. Desde a fundação houve o empenho de estratégias jornalísticas e de *marketing* que permitiram a ambas as publicações se tornarem referência entre a comunidade científica e o público não especialista. Ambas as revistas foram consideradas por Barata publicações híbridas, com características de periódicos científicos e de revistas de divulgação de ciência.

Revistas de prestígio internacional como essas lideram os debates sobre prioridades no meio científico mundial e, portanto, são responsáveis pela legitimação da ciência e contribuem para fundamentar as atividades da comunidade científica nos chamados paradigmas dominantes (Kuhn, 1997). Trata-se de periódicos científicos que estabelecem padrões e inspiram tendências para outras publicações, tais como o modelo de revisão por pares, a formatação, os critérios rigorosos para aprovação de artigos, a presença na mídia, a comunicação com o público e outros aspectos da editoração científica. A credibilidade desses periódicos internacionais perante a comunidade acadêmica internacional reforça o domínio de países desenvolvidos no cenário de produção científica mundial.

Dados do relatório *Analysis of bibliometric indicators for European policies 2000-2013* (European Commission, 2015), a partir da análise de indicadores bibliométricos para medir a quantidade e o impacto de publicações científicas em várias partes do mundo, concluíram que os EUA, por exemplo, publicaram, no período estudado, 23% dos artigos indexados na base Scopus (cerca de 19,5 mil títulos de mais de cinco mil editoras internacionais, incluindo a cobertura de 16.500 revistas com revisão por pares nos campos científico, técnico e de ciências médicas e sociais), seguidos pelo Reino Unido (5% dos artigos indexados). Ambas as nações seguiram nas primeiras posições da produção científica mundial até 2017, quando a China conquistou a liderança (White, 2019). Com o aumento de citações de artigos de revistas científicas chinesas, consideradas um indicador de qualidade das publicações, é esperado que cresça também a competição por visibilidade e prestígio.

A prática da divulgação científica também tem sido uma oportunidade de expandir a missão das revistas científicas para se comunicar não apenas para especialistas, como era o propósito dos periódicos desde sua origem, em 1665, mas também para interessados de outras áreas do conhecimento e não especialistas. No Brasil, há estudos que analisam as revistas científicas sob o ponto de vista da altmetria,^2^ portanto, o modo como essas publicações atuam na divulgação de conteúdos para a sociedade (Araújo, 2015; Gouveia, 2016). As estratégias usadas pelas publicações científicas brasileiras e o potencial da comunicação extramuros da academia têm se mostrado vantajosos para atrair citações, visibilidade e novos públicos, mesmo quando os recursos e a equipe são limitados.

Este artigo analisa as estratégias de comunicação de duas revistas médicas internacionais centenárias, a estadunidense *NEJM* (de 1812) e a inglesa *The Lancet* (de 1823). Ambas as publicações possuem grande prestígio na comunidade científica internacional, liderando *rankings* de indicadores de impacto científico entre as revistas da área médica, como o Fator de Impacto (FI).^3^ Essas revistas têm conquistado FI crescentes desde 2000, atingindo níveis extremamente altos no pós-pandemia. *The Lancet*, por exemplo, saiu de um FI 10,232 em 2000 para 168,9 em 2022; enquanto a *NEJM* cresceu de 29,512 em 2000 para 158,6 em 2022.^4^ Apesar de serem revistas majoritariamente de acesso restrito (apenas para assinantes), elas concentram, cada vez mais, as citações e, portanto, o prestígio na comunidade médica mundial. Parte desse prestígio parece ser acentuado por esforços contínuos em manter a visibilidade por meio das estratégias de comunicação (Grady, Wetterlow, 1978).

Nossa análise se pauta no papel da comunicação para o público não especializado nas revistas médicas *NEJM* e *The Lancet*, a partir das trajetórias históricas de ambas para identificar como a comunicação foi incorporada às políticas.

Assim, colocam-se as seguintes perguntas de pesquisa:

1. Historicamente, qual lugar a divulgação da medicina para o público não especializado ocupa em duas revistas médicas de grande prestígio mundial, *The Lancet* e *NEJM*?
2. A divulgação para o público não especializado ganhou relevância ao longo da história das revistas médicas?

Para responder às perguntas de pesquisa, analisaremos as trajetórias editoriais e as estratégias de divulgação das revistas científicas *NEJM* e *The Lancet*.

## Metodologia

O *corpus* deste artigo é composto por 47 publicações de ambas as revistas médicas, incluindo os “comemorativos” (assim chamados os artigos publicados nos meses de aniversário de publicação), sobre mudanças editoriais relacionadas às estratégias de comunicação, sendo 19 da *NEJM*, e 28 de *The Lancet* (veja material suplementar em Barata, Medeiros, 2025).

Neste estudo, utilizaram-se as ferramentas de busca das próprias revistas para localizar editoriais comemorativos ou relacionados à comunicação e à divulgação científica. A busca por palavras-chave no indexador WoS^5^ mostrou-se pouco eficiente, uma vez que essa ferramenta não diferencia editoriais de artigos ou outros formatos. Optou-se pelo levantamento por busca de arquivos de ambas as revistas. A busca pelos editoriais comemorativos foi realizada a cada cinco anos, na data de lançamento de cada revista. As palavras-chave usadas foram: anniversary OR celebration OR celebrate AND communication OR journalism.

Realizou-se uma busca complementar em outras publicações (comentários, artigos, revisões e notícias) mencionadas na primeira amostra, além de publicações de outros periódicos.

Procedeu-se à análise dos conteúdos para identificar as principais mudanças nas linhas editoriais e os investimentos em equipe e as estratégias de comunicação adotadas.

### The New England Journal of Medicine (NEJM)

A primeira edição da revista *The New England Journal of Medicine and Surgery and the Collateral Branches of Science* foi publicada em 1º de janeiro de 1812, em Boston, nos EUA, com frequência trimestral (Podolsky, Greene, Jones, 2012, p.1457). Ela surgiu 15 anos após o aparecimento da primeira revista estadunidense – *The Medical Repository*, criada em Nova York, em 1797 (Green, 1923). Em 1828 foi incorporada à revista *The Medical Intelligencer*, passando a se chamar *The Boston Medical and Surgical Journal* (Garland, 1928)*.* Em 1921, foi novamente vendida, passando para a responsabilidade da Massachusetts Medical Society, da qual recebeu financiamento e sustentabilidade financeira, e, em 1927, foi renomeada como *The New England Journal of Medicine* (Garland, 1928).

Em sua origem, a *NEJM* tinha como objetivo dialogar com alunos da área médica, uma vez que não havia no país um periódico que cumprisse essa missão, e hoje é considerada uma das revistas médicas de maior prestígio internacional, como indica o alto número de citações (o FI em 2022 foi de 158,5) e a atenção social que seus artigos recebem (está sempre entre as cinco ou dez revistas científicas com maior pontuação de atenção social, segundo o Altmetric Attention Score).

Em editorial comemorativo, Allan M. Brandt (2012), historiador de medicina da Universidade de Harvard, menciona que, durante os 200 anos de circulação, além da evolução estrutural, o periódico sempre manteve a preocupação em publicar grandes descobertas médicas e estudos relacionados ao ensino médico norte-americano, em especial o praticado nos EUA (Campion et al., 2010).

Desde sua criação, manteve-se firme no propósito de dialogar com os profissionais da área médica (Fishiben, 1928).

A *NEJM* seguia uma tradição de revistas médicas iniciada pela francesa *Nouvelles Découvert sur Toutes les Parties de la Médecine* (lançada em 1679 e encerrada em 1681), seguida pela *Medicina Curiosa*, primeira publicação inglesa, em 1684 (Fishbein, 1928), por *The Medical Repository*, lançada em 1797, por *The Philadelphia Medical Museum*, criada em 1804, como a primeira revista médica estadunidense lançada em Nova York, além de outros periódicos na industrializada região de Boston, local de cursos de medicina. Fishbein ainda atribui o mérito da primeira publicação médica semanal a *The Medical Intelligencer*, em 1823, igualmente em Boston, mesmo ano em que *The Lancet* foi criada em Londres, também com publicações semanais.

Em 1810, o nordeste dos EUA já tinha seis escolas de medicina com cerca de 1.375 estudantes; população que chegaria a 4.338 dez anos mais tarde (Fishbein, 1928). Os cursos de medicina respondiam à demanda de uma região populosa com grande fluxo migratório que favorecia surtos epidêmicos de tuberculose e varíola, apesar de já existir vacina, além de à pressão por uma formação genuinamente norte-americana e menos dependente da Inglaterra. Nesse contexto, as revistas médicas eram um meio de circulação da informação de modo rápido, com atualização nacional e internacional, para profissionais e estudantes, e, portanto, constituíam-se como uma maneira de fortalecer a comunidade médica e a medicina nacionais. Metade da população médica não era bem formada (Fishbein, 1928). Logo, os periódicos também fomentaram a educação de médicos não acadêmicos e novos padrões de redação e rigor científico. Fishbein chama de jornalismo médico o que os editores de revistas médicas semanais praticavam em *JAMA* e *The Boston Medical and Surgical Journal* (ou *NEJM*).

No âmbito da estrutura, a primeira grande mudança da revista aconteceu em 1828, quando o periódico, que passava por dificuldades financeiras, foi incorporado a *The Medical Intelligencer* para formar o semanal *The Boston Medical and Surgical Journal* (Rothstein, 1987). O editorial afirmava que,

neste centenário, o periódico se destaca como o mais antigo representante do jornalismo médico na Nova Inglaterra, e uma possível exceção na América, com um futuro auspicioso que deve continuar a incorporar o caráter e o progresso científico da profissão na Nova Inglaterra (Garland, 1928, p.1).^6^

Os 50 anos da revista foram comemorados a partir da fusão em 19 de fevereiro de 1828, período em que existiam apenas oito revistas médicas nos EUA (Billings, 1879). Apesar de haver 54 revistas médicas no país em 1879, só outras duas tinham idade igual ou superior à revista quinquenal, ambas da Filadélfia. Surpreendentemente, indicando enorme produtividade, em relação ao total de 372 revistas médicas no mundo. O editorial de Billings evidencia que o termo “jornalismo” é empregado para descrever a prática de redação médica do editor, com liberdade para expressar sua opinião e crítica.

Em 1873, a revista passou a ser semanal com máximo de cinco artigos, passando para 12 (Kassirer, 1999). No final do século XIX, o número de artigos científicos por edição aumentou para 15; em seguida, a mudança ocorreu na avaliação dos artigos, que começou a ser realizada por mais de um especialista, dando início ao trabalho de revisão por pares na revista (Fishbein, 1928).

No ano do primeiro centenário da revista, ainda intitulada *The Boston Medical and Surgical Journal*, o editorial expressou a preocupação com anúncios que mimetizavam notícias médicas nos jornais e que, muitas vezes, iludiam o público desinformado sobre tratamentos sem comprovação médica ou com potenciais danos à saúde dos leitores (Garland, 1928). O conflito entre publicidade e autonomia da publicação retornaria à própria *NEJM* nos anos 1950.

Em 1999, a demissão do editor da *JAMA* reacendeu o debate sobre a importância de os editores de revistas científicas e a mídia terem autonomia com relação às decisões e opiniões dos proprietários dos periódicos ou das associações médicas (Campion et al., 2010). A demissão trouxe para a arena não apenas uma crítica de editores de outras revistas médicas, como a *NEJM*, mas a influência de revistas médicas, como a *JAMA*, nas políticas públicas.

Em 1921, a *NEJM* passou a fazer parte da Massachusetts Medical Society, uma tendência em busca de apoio de sociedades científicas que inúmeros outros periódicos seguiram para garantir a estabilidade financeira, por um lado, e a visibilidade, por outro. Sobre o apoio de recursos que essa sociedade passou a investir na revista estava a defesa, entre inúmeros fatores, de que a sociedade se empenhasse adequadamente da maneira mais ampla no sentido de trazer as conquistas da medicina para a atenção de todos os médicos praticantes e disseminar o conhecimento das instalações de hospitais para instrução de alunos, bem como o tratamento dos enfermos, indo ao encontro da missão da instituição, que preconiza a saúde como essencialmente necessária para a felicidade da sociedade (Brandt, 2012).

Durante o século XX, cresceu a competição entre revistas médicas, mas também entre elas e o jornalismo. A preocupação em resguardar a informação médica levou o editor Franz J. Ingelfinger a estabelecer uma das mudanças mais significativas na cobertura médica. Em 1969, a chamada regra de Ingelfinger estabeleceu o direito da revista de publicar apenas artigos inéditos, isto é, que não tivessem sido divulgados em nenhum outro veículo, incluindo órgãos de imprensa (NEJM, 1969; Relman, 1981). A regra surgiu após uma série de críticas que o então editor recebeu quando se recusou a publicar um relatório programado para a revista, mas publicado antes pelo *Medical World News* (Relman, 1981).

Os críticos dizem que a regra restringe o livre fluxo de informações, enquanto os proponentes afirmam que as informações de um artigo divulgado antecipadamente podem ser imprecisas porque o artigo não foi submetido à revisão por pares (Altman, 1996, p.1382).

A regra, posteriormente adotada por outras revistas científicas, foi uma forma de a revista ter exclusividade sobre o conteúdo nela publicado, mas Ingelfinger também acreditava que os médicos ocupavam a melhor posição no que diz respeito à capacidade de avaliar a qualidade dos artigos médicos, e que a divulgação prematura na mídia da pesquisa médica e de trabalhos ainda não documentados poderia causar confusão entre os leigos. “Acreditamos firmemente na importância de um público informado e no direito do público de saber, mas também acreditamos que é importante ganhar tempo para garantir que o público não esteja sendo enganado” (Relman, 1981, p.825).

Diante dessa preocupação, a revista reconhecia e legitimava a necessidade e a importância do trabalho da mídia, uma vez que, na visão de seus editores, “a imprensa popular pode desempenhar um papel muito importante no relato e na explicação dos novos desenvolvimentos para o público” (Relman, 1981, p.825).

Apesar da defesa do trabalho jornalístico da grande mídia, a revista se considerava apta a publicar, além de evidências científicas, também notícias sobre novas descobertas científicas, não se restringindo aos textos técnicos e ampliando, assim, o número de leitores (Grady, Wetterlow, 1978) e, com isso, também garantia a exclusividade e a sobrevivência da revista médica em um mercado competitivo.

Em 1977, a *NEJM* se tornou a primeira revista científica a receber o prêmio George Polk Award for Journalistic Excellence na categoria de Reportagem sobre Ciência (Science Reporting) (NEJM, 1978; Campion et al., 2010), indicando sua relevância na divulgação da medicina para o público não especializado.

Dez anos mais tarde, ao celebrar os 175 anos da revista, o editorial de Arnold S. Relman (1987) compartilhou as inovações técnicas que melhoraram a qualidade das cores nas ilustrações e o valor das publicações. Ele também chamou atenção para a importância de a revista compartilhar seus conteúdos com a imprensa, que recebia os estudos antecipadamente, para poder divulgar às quintas-feiras, junto com a publicação da revista (Kassirer, 1999). A comunicação da revista ganharia fôlego e visibilidade com o desenvolvimento da internet e das redes sociais, espaços nos quais segue atuando hoje de forma intensa.

### NEJM no ambiente digital

A revista investe pesadamente em redes sociais, com conteúdo ágil e variado, o que resulta em elevada interação, algo perceptível tanto na página quanto nas redes. Assim, a *NEJM* se consolidou como uma das mais importantes revistas científicas da área médica, reforçando sua missão: abordar assuntos de interesse social, sem deixar de lado o diálogo com os profissionais da área. E o resultado é percebido na conquista de índices significativos no que diz respeito ao FI, à altmetria e aos seguidores em redes sociais. Esse trabalho intenso no ambiente *on-line* teve início em 1996, com o lançamento do seu primeiro *website*, no qual eram publicados apenas resumos dos artigos presentes na versão impressa da revista, e sua inserção era feita de forma manual, com periodicidade semanal (Lancet, 2012).

O *website* da *NEJM* passou a publicar artigos com antecipação de sete a nove semanas do exemplar impresso. A medida era para garantir que artigos com impacto na prática médica estivessem disponíveis rapidamente (Brandt, 2012). “Pretendemos aproveitar ao máximo a nova tecnologia para melhorar a forma como a revista comunica as informações médicas a seus leitores” (Campion et al., 2010, p.1175).

Já no início dos anos 2000, mais do que reproduzir os artigos impressos, o *site* começou a contar com um novo formato de comunicação com os leitores: os vídeos, um meio que, na visão dos editores da revista, é altamente eficaz para comunicar informações biomédicas complexas de uma forma que a imprensa não tem condições de fazer, por ser limitada ao texto e à imagem fixa (Brandt, 2012).

Em 2007, os artigos, suas imagens e os vídeos explicativos ganharam um novo espaço: os canais de mídia social Facebook e Twitter (atual X). Em novembro de 2024, a página da *NEJM* no X contava com mais de 945 mil seguidores, no perfil principal, e a do Facebook, com mais de 1,8 milhão de seguidores, e 553 mil no Instagram.

Segundo dados do próprio *site* da revista, semanalmente cerca de um milhão de leitores recebem o sumário da revista por *e-mail* (por meio dos *mailings* de agências de notícias), e a cada mês cerca de 2,5 milhões de usuários se conectam eletronicamente ao *site* do periódico. A edição digital da revista também possibilitou o acesso restrito – alguns artigos são de acesso aberto – para usuários de mais de cem países (Campion et al., 2010).

Em 2012*,* seguindo uma tendência de outras revistas científicas – *Cell*, *Nature*, *Science* e *The Lancet* –, foi criado o Group *NEJM*, que incorporou a revista inglesa, o *NEJM Journal Watch –* considerada a “voz independente dentro do grupo” (Lancet, 2024), pois se trata de um *blog* independente do *site* da revista no qual autores e editores médicos fazem a curadoria e resumem pesquisas de mais de 250 periódicos médicos, bem como de outras fontes de notícias médicas. Posteriormente, o *NEJM Catalyst* foi criado com o objetivo de “reunir profissionais da área médica, líderes clínicos e médicos para compartilhar ideias inovadoras e aplicações práticas para melhorar a prestação de cuidados de saúde” (Lancet, 2024).

Em seguida, foram criados o *NEJM Knowledge+* – seção que reúne perguntas de leitores que são respondidas por mais trezentos autores e editores – e o *NEJM Career Center* – um conjunto de ferramentas de busca de empregos para médicos, além de artigos sobre a carreira médica e as tendências de emprego. Há ainda a seção “Image Challenge” [“Desafio da Imagem”], que traz semanalmente um desafio de uma foto de um paciente com a descrição de sintomas com os quais o público deve interagir, mas claramente com um apelo para estudantes de medicina, que, depois, acessam o diagnóstico correto. A equipe de edição da revista é dividida em oito grupos de trabalho: editores, conselho editorial, sistemas editoriais (atuam nas revistas do Group *NEJM*), grupo editorial *on-line*, edição de manuscrito, artes gráficas, tecnologia de produção editorial (edição de todo conteúdo em formato atraente), e grupo de perspectivas (verifica se o conteúdo está dentro do escopo) (Müller, Duff, Stern, 2012).

Por ocasião das comemorações do bicentenário da revista, Kohane, Drazen e Campion (2012, p.2539) previram que, nos próximos cem anos, “as sociedades aceitarão que o conhecimento abrangente sobre doença, prevenção e tratamento eficaz é um bem público essencial”, e bastou menos de uma década para que essa afirmação se tornasse tão crucial em meio à pandemia da covid-19. Eles acrescentam que “o poder crescente das tecnologias de informação e comunicação pode ajudar a encontrar maneiras de melhorar a saúde global” (p.2539).

Ainda em comemoração aos 200 anos de publicação, a revista reforçou sua missão: “Nosso foco continua em toda a comunidade médica, com seus conhecimentos científicos e avanços nos cuidados clínicos” (NEJM, 2012, p.383). Porém, em 2019, com a mudança do editor-chefe, a revista reafirmou seu compromisso: “Afinal de contas, nossa missão não é realmente publicar uma revista científica. É, em vez disso, comunicar as informações médicas mais importantes aos provedores e seus pacientes” (Rubin, 2019, p.1069). E um público tão variado exige publicações com linguagem e formatos diferenciados para facilitar a compreensão dos conteúdos e uma leitura mais ágil de quem busca se atualizar sobre sua área profissional, medicina e saúde de uma forma geral.

### The Lancet

No século XIX, o Império britânico experimentou um período de intensa produção cultural e artística. Em contrapartida, a medicina profissionalizante se mostrava ainda incipiente e seguia não regulamentada, assim como os medicamentos, e os médicos lutavam contra doenças sobre as quais pouco sabiam e cujos tratamentos eram pouco efetivos. Essa época também ficou marcada pela precariedade da cirurgia, a ausência da anestesia e a falta de higiene.

Foi nesse cenário de caos no campo médico que o cirurgião britânico Thomas Wakley (1795-1862) fundou, em 5 de outubro de 1823, a revista médica *The Lancet*, com o propósito de abordar em suas páginas aquela que era a maior bandeira política: a reforma do sistema médico no Reino Unido (Lancet, 1923). Aconselhado e influenciado pelo amigo e jornalista político radical William Cobbett, Wakley via a escrita como a melhor arma para atacar más condutas médicas (Jones, 2009; Lancet, 1923). “Wakley tornou-se cada vez mais ciente da corrupção generalizada dentro da profissão médica, na qual a influência contava mais do que a capacidade, e a ignorância e a avareza estavam desenfreadas” (Jones, 2009, p.406).

O primeiro editorial já prometia “transmitir ao público e aos profissionais distantes, bem como a estudantes de medicina e cirurgia, relatos de palestras de hospitais metropolitanos” (Lancet, 1823, p.1). No centenário, o editorial reforçava que, desde a origem, “o conteúdo foi nominalmente endereçado, tanto para o público como para o leitor médico, [para o qual] pressupunha-se familiaridade com as situações profissionais” (Lancet, 1923, p.698).

Em 1832, Wakley concorreu ao Parlamento pela primeira vez e perdeu; tentou novamente em 1835, ganhou e permaneceu no cargo por 17 anos. Ele foi um dos responsáveis pelo conteúdo do Ato Médico de 1858, no qual foi estabelecido o Conselho Geral de Educação e Registro Médico (Jones, 2009, p.405). Ao longo desse período, lutou pela reforma da medicina forense, pela saúde pública e por outras questões médicas e fez campanha para reinventar o ofício do médico-legista como uma instituição aberta, democrática e medicamente competente. Outra causa defendida fortemente por Wakley era a reforma sanitária inglesa, como visto em um editorial de 1862, no qual o médico descreve os problemas de saúde enfrentados pelos ingleses em decorrência da falta de saneamento básico, uma realidade bem diferente da que existia no Castelo de Windsor, já naquela época (Jones, 2009).

Desde a primeira edição de *The Lancet*, em 1823, a revista objetivava disseminar informação médica confiável, atual e relevante para médicos pesquisadores e práticos, estudantes de medicina e indivíduos em geral. As aulas-palestras do médico Astley Paston Cooper e de outros renomados profissionais tornaram-se um marco (Lancet, 1823). Wakley incomodava por sua postura crítica, muitas vezes denunciava instituições médicas, políticas de saúde pública, escolas de medicina e questões sobre a própria comunidade médica, o que rendeu a publicação de inúmeros processos de difamação, já na primeira década de existência (Garner, 1973). A atuação de Wakley e de *The Lancet* contribuiu para aperfeiçoar a medicina do início do século XIX (Garner, 1973), a publicação científica e o jornalismo médico (Jones, 2009).

Segundo Conrad Keating (2023), médico da Escola de Medicina, do Trinity College Dublin, muitas das descobertas da área médica, como ensaios clínicos e estudos epidemiológicos – que analisam os determinantes da saúde e da doença entre indivíduos e sociedade – foram relatados pela primeira vez na revista inglesa. Outros autores reforçam a missão da revista não apenas nos aspectos técnico-científicos, mas também de políticas médicas.

Desde os primeiros dias de *The Lancet*, há 200 anos, a cirurgia teve um papel central na sua existência. A revista tem funcionado como um centro nevrálgico, ligando a inovação cirúrgica por parte dos indivíduos com o desafio e o debate na profissão em geral (Frampton, Kneebone, 2023, p.1222).

Outro artigo de opinião que destaca a “relação” da revista inglesa com a sociedade foi enfatizado em “*The Lancet* and the medical journal as an instrument of reform” (Podolsky, 2023, p.1220), que tratou da identidade da revista: um instrumento de reforma na medicina e na sociedade e, crucialmente, na redefinição das próprias fronteiras entre ambas.

É, contudo, o editorial “*The Lancet* at 200 years: timeless mission to drive positive social change through advancing medical research and science” (Lancet, 2023, p.1200) que enfatiza a missão social da revista:

A *Lancet* foi fundada em 1823 por Thomas Wakley com a visão de impulsionar mudanças sociais positivas por meio do avanço da investigação médica e da ciência para um bem maior, abordando as desigualdades da época com acesso ao conhecimento médico para ‘profissionais médicos e cirúrgicos’ no Reino Unido e nas colônias britânicas (como eram chamadas na época) e por ser mais do que uma revista médica.

Wakley permaneceu como editor do periódico até sua morte, em 1862, quando assumiu o cargo seu filho Thomas Wakley Filho, que, após se formar em medicina, deu prosseguimento à batalha do estudante de medicina (Lancet, 1923, p.700). Wakley Filho se valeu das páginas da revista para fazer denúncias de situações em que a lei era distorcida, mas sem deixar de dialogar com os médicos. Esse interesse em promover mudanças no sistema de saúde inglês, bem como na saúde pública do país, ficou estampado nas páginas das edições do século XIX, como em 1º de outubro de 1831, quando a revista dedicou uma edição à epidemia de cólera que assolava a Europa na época e um número especial sobre tratamentos alternativos para pacientes com demência (Lancet, 1923).

Vale destacar que um período-chave na história de *The Lancet* foi alcançado em 1850, quando a revista tinha se estabelecido há mais de um quarto de século e gozava de reputação de solidez, franqueza e grande esforço, que se espalhou sobre o país e sobre o continente (Lancet, 1923).

Em 1973, quando completava 150 anos, a *Canadian Medical Association Journal* dedicou o editorial de sua 150ª edição a expor sua opinião acerca do papel de *The Lancet* desde sua criação. Já em 1972, o Instituto Americano para Informação Científica tabulou as cinquenta revistas científicas mais citadas no mundo, e a revista inglesa ocupou a 11ª posição do *ranking*.

As edições de *The Lancet* de 1923, como forma de comemorar o centenário da revista, destacaram em seus editoriais os temas que despertavam mais interesse por parte dos leitores. Entre eles, as primeiras experiências com nutrição – os atos da anatomia, a sofisticação dos alimentos, a conduta médica e a reforma do sistema de saúde inglês promovidas pelo Relatório Dawson^7^ (Lancet, 1923, p.700).

O relatório estabeleceu diretrizes que influenciaram, de maneira significativa, o sistema nacional britânico em 1948, que passou a orientar a reorganização dos sistemas de saúde em todo o mundo (Lancet, 1923).

Sobre esse tema, o editorial “Medicine and the public” (Lancet, 1923) recupera a primeira tentativa feita por Wakley de entrar no Parlamento inglês e, com isso, a mudança significativa nas páginas da revista: o caráter. Não houve esforço para difundir informações gerais, mas, sim, esforço para mostrar a universalidade da medicina em si, permeando todas as circunstâncias e atividades da vida.

Pode-se dizer que a revista construiu ao longo do século XIX uma imagem de articulação e pioneirismo, ao dialogar com médicos e estudantes de medicina e ao retratar uma sociedade que ansiava por mudanças em seu sistema de saúde.

Com a virada do século e a mudança do sistema de saúde inglês, artigos sobre estudos pioneiros envolvendo doenças que assolavam a humanidade em diversos países passaram a ganhar destaque nas páginas de *The Lancet*; entre elas malária e febre amarela. É nessa época também que a patologia clínica e a saúde pública começam a ganhar espaço na revista inglesa.

A revisão por pares foi debatida nas páginas de *The Lancet* em 1993, no artigo “Early announcements”, no qual a revista argumentava que a divulgação precoce dos resultados de um ensaio clínico (antes da revisão por pares) poderia gerar informações distorcidas. Essa visão era também defendida pelo Comitê Internacional de Editores de Revistas Médicas, do qual a revista inglesa fazia parte (Lancet, 1993).

Já a relação entre ciência e mídia pautou outros editoriais e artigos ao longo da história da revista; dentre eles merece destaque o artigo de Fiona Fox (2014), no qual ela discute as razões pelas quais cientistas deveriam envolver a mídia em suas pesquisas. Para a autora, a maioria dos cientistas é financiada com recursos públicos; a população, portanto, tem o direito de saber como esse dinheiro está sendo gasto e para onde os cientistas estão caminhando, argumento ainda usado nos dias de hoje. Ela também aponta ser uma boa oportunidade de colocar a ciência em domínio público no exato momento em que o público em geral realmente se importa.

Há ainda o texto “A health scare in the mass media”, no qual a revista discute a questão do embargo de novos estudos e também o conhecimento prévio do jornalista em áreas específicas da ciência (como a epidemiologia, por exemplo) na tentativa de evitar a má divulgação de descobertas do campo da saúde nos meios de comunicação, assunto que preocupava cientistas e médicos (Lancet, 2002).

Porém, não apenas os temas dos artigos mudaram, mas o volume de artigos tornou-se maior a cada edição. As edições ganharam editorias, fazendo com que os artigos fossem dispostos por categorias: editorial, estudos clínicos, saúde pública e comentários e notícias. O século XXI ficou marcado ainda por outros artigos de impacto que, assim como no século anterior, retratavam a sociedade da época, proporcionando à revista o *status* de “uma das principais revistas médicas da atualidade” (Jones, 2009, p.404).

Em outubro de 2010, reuniram-se evidências sobre como os meios de comunicação de massa podiam influenciar a saúde, reforçando, assim, a preocupação que a revista sempre demonstrou com a comunicação dirigida aos pares e não especialistas. O debate em questão era sobre a exposição de jovens a comportamentos pouco saudáveis por meio de diferentes canais de comunicação.

No início de 2022, ano que antecede os 200 anos de publicação ininterrupta, a revista inglesa publicou o editorial “The state of science and society in 2022”, um debate sobre o quanto a ciência tem sido frequentemente atacada. Em meio à pandemia de covid-19, a revista reuniu fatos, relatos e resultados de pesquisas que apontaram o quanto a retórica anticiência ganhou força ao redor do mundo, levando cientistas a sofrer com ameaças de diferentes naturezas e o quanto esse discurso foi prejudicial na condução da pandemia em muitos países, em especial no Brasil (Lancet, 2022).

Uma análise do acervo permite apontar que *The Lancet*, ao longo de quase 200 anos, acompanhou as transformações sociais e tecnológicas da sociedade como um todo. Um exemplo disso foi a mudança na estrutura da revista em relação ao processo de revisão por pares. Hoje, esse modelo ainda permanece, porém, divide espaço com outro, denominado *fast track*. Neste último, bastante usado por publicações da área de saúde, ocorre uma espécie de aceleração da etapa de revisão dos artigos e, às vezes, também dos demais processos, chegando ao encurtamento do tempo de publicação (Lancet, 2015).

Dados os critérios de qualidade de indexadores internacionais para publicações acadêmicas *on-line*, o ideal é que todo o processo de avaliação dos artigos por parte dos periódicos não ultrapasse o prazo de noventa dias. Na modalidade *fast track* esse prazo pode cair para 28 dias ou menos e a revisão por pares pode ser feita em 72 horas.^8^ A principal justificativa das revistas adeptas desse modo acelerado de avaliação é dar rápida visibilidade a artigos que apresentem grande contribuição a sua área e potencial de implicações práticas imediatas.

### The Lancet no ambiente digital

Um marco importante na estrutura da revista se deu no início dos anos 1990, com o advento da internet, quando *The Lancet*, além das edições impressas, criou seu primeiro *site*, digitalizando as edições dos últimos 100 anos e disponibilizando-as em seu endereço eletrônico, além da publicação de resumos de artigos presentes na versão impressa da revista. A inserção dos conteúdos era feita de forma manual com periodicidade semanal (Lancet, 2012).

Segundo o *site* atual da revista, *The Lancet* conta com equipe própria para a manutenção das redes sociais, o que é chamado pela revista de “Grupo editorial *on-line*” (*Online editorial group*), sendo composto por profissionais especialistas em conteúdo eletrônico e produção na *web* (Lancet, 2012). Foi somente em 2010 – quando o *site* passou por uma reformulação e ganhou o formato atual – que o acervo da revista foi ali inteiramente disponibilizado mediante pagamento. Desde então, o leitor tem acesso ao acervo digitalizado da revista e a todas as revistas da “família *The Lancet*”, além dos artigos da edição em circulação da revista principal e de um vasto material de divulgação, como descrito no *site* da revista.

Se o foco da revista é ser um importante canal de informação médica e acompanhar as novas tendências da sociedade, a inserção nas redes sociais também integra o espectro de mudanças pelas quais *The Lancet* passou no século XX. Embora em 2008 já se pensasse em redes sociais, somente em 2010 a revista se consolidou no Facebook e, em 2021, no atual X, que passaram a disponibilizar de forma mais rápida o conteúdo interativo produzido pela equipe da revista.

Em novembro de 2024, a página da revista no X se mostrou a mais popular, com 749 mil seguidores, enquanto no Facebook, em página com destaque para o *slogan* “More than a Medical Journal” (“Mais do que uma revista médica”), eram 356 mil seguidores, e com menor presença no Instagram, com pouco mais de 18 mil seguidores. Esses canais fazem uso de material escrito e audiovisual para abordar de maneira simples e objetiva os diferentes temas publicados nas revistas da “família *The Lancet*”.

A equipe da revista é composta pelos editores da revista, *ombudsmen*, revisores, destacando que a publicação conta ainda com um departamento especializado em comunicação, ou seja, profissionais especializados em produção de conteúdo para redes sociais, agências de notícias e jornalistas.

Em 2015, outra mudança significativa passou a vigorar na revista inglesa, a inserção do “Research in Context”, material que juntamente com outros itens tornou-se indispensável durante a submissão de um artigo na revista. Esse texto, como define a própria revista em seu *site*, “deve incluir uma descrição de todas as evidências que os autores consideraram antes de executar este estudo” (Lancet, 2015).

A página da revista no X reúne o trabalho de divulgação de novos artigos, edições e assuntos potenciais para pautar mudanças no campo das políticas em saúde. A revista tem como público-alvo os profissionais do campo médico, dada sua forte preocupação em divulgar e fomentar políticas públicas e trazer ao público menos especializado assuntos da área da saúde pública. O Altmetric, que mede a *performance* dos artigos em plataformas digitais, indicou que o X vem desempenhando papel importante para além dos muros das universidades, o que pode indicar investimento das revistas *The Lancet* e *NEJM* nessa rede social que, apesar de pouco numerosa em usuários, se comparada a outras redes sociais, concentra usuários que influenciam a opinião pública, como políticos, jornalistas, cientistas e influenciadores, considerados estratégicos.

Pacientes usam as redes sociais para pesquisar seus sintomas, seus médicos, seus tratamentos e para obter informações de grupos de apoio. Médicos podem usar redes sociais para conscientização, para fornecer informação precisa e como um portal de comunicação com outros profissionais da saúde (Lancet, 2012, p.1562).

## Discussão

Este artigo reúne os resultados da análise do histórico das revistas científicas *NEJM* e *The Lancet*, a fim de entender a relação da revista com comunicação para a sociedade como um todo, bem como apontar as principais práticas dessas revistas para divulgar seus artigos e o impacto gerado por meio dessas práticas de comunicação.

A revisão bibliográfica de artigos científicos e principalmente de editoriais das próprias revistas permitiu entender esse processo, que tornou possível afirmar que a comunicação, para além dos especialistas da área, sempre fez parte do universo das duas publicações. Um exemplo que merece destaque, no caso da *NEJM*, é a declaração do então editor Arnold S. Relman, no editorial de 1º de outubro de 1981, no qual ele reforça que o papel dos jornais é diferente do papel das revistas científicas, e que jornais só devem publicar as “novidades” da ciência simultaneamente ou após divulgadas pelas revistas especializadas. O editor enfatiza sua preocupação com a qualidade da cobertura feita pela mídia dos artigos científicos da época; para ele, muitos detalhes eram deixados de lado (Relman, 1981, p.824).

Um aspecto de grande relevância em *The Lancet* é que, já no primeiro editorial, o então editor, Thomas Wakley, explicita o papel que a revista pretendia desempenhar (e que desempenha até hoje): promover, por meio dos artigos científicos, o debate sobre assuntos relacionados às políticas de saúde do Reino Unido, divulgar as grandes descobertas científicas e fomentar a promoção do diálogo com estudantes de medicina. A revista ampliou sua atuação, passando a ser uma importante voz e incentivadora de políticas de saúde pública pelo mundo. Exemplo disso é a iniciativa *The Lancet* Countdown, organizada pela revista *The Lancet* e pela Wellcome Trust, desde 2016, para garantir a implementação de políticas de saúde pública em função dos impactos causados pelas mudanças climáticas em várias regiões do planeta.

Embora, desde os lançamentos, as duas revistas científicas tenham públicos-alvo distintos, ambas se preocuparam em buscar e assegurar o diálogo com outros públicos, além dos cientistas. Antes do advento da internet, como visto ao longo desta análise, esse trabalho era feito por meio de eventos científicos e boletins informativos. Com a internet, e em especial as redes sociais, esse trabalho ganhou ainda mais força e significado dentro das duas revistas científicas.

Ao longo dos primeiros anos de publicação, falar de ciência em uma linguagem acessível a todos os públicos era algo que acontecia por meio dos próprios editoriais das revistas. Esses apresentavam, de forma objetiva, as principais informações dos artigos de destaque daquela edição, ou seja, não era apenas uma apresentação do que o leitor iria encontrar naquela edição, mas explicações em linguagem acessível sobre temas muitas vezes complexos para quem não era da área médica. Outro caminho encontrado pelas publicações para tratar de forma acessível o conteúdo de suas novas edições era por meio do diálogo com jornalistas.

Tanto *NEJM* quanto *The Lancet*, porém, fizeram ressalvas à prática jornalística, o que motivou esforços no sentido de realizar a divulgação de seus conteúdos na tentativa de minimizar discursos equivocados sobre as pesquisas publicadas em suas páginas. Com isso, as revistas passaram a produzir materiais e os enviar aos jornalistas para que fossem divulgados à sociedade. Trabalho esse que, desde o advento da internet, passou a ser feito por meio de *press releases* das agências de notícias, mas também diretamente por canais de comunicação das próprias revistas.

Os indicadores de atenção social da ciência, medidos pela altmetria em dados do Altmetric.com, mostram que, entre os cem artigos científicos divulgados pela mídia internacional entre 2013 e 2020, tanto *The Lancet* quanto *NEJM* tiveram grande visibilidade em plataformas digitais, levando os resultados de seus estudos para além do público acadêmico. Esse é um esforço dessas e de outras revistas científicas em investir na comunicação pública da ciência, com mais visibilidade, atentas ao interesse público pela temática de saúde e medicina – o que acaba tendo forte relação com a credibilidade científica conquistada com essa exposição.

## Considerações finais

As trajetórias editoriais e as estratégias de divulgação das revistas científicas *NEJM* e *The Lancet* mostram que a divulgação esteve sempre presente em suas preocupações de alcançar o público além do cientista e como ferramenta fundamental nos cotidianos editoriais. Cada vez mais, com as redes sociais e a comunicação direta com a sociedade, sem a moderação de jornalistas, as revistas atuam com estratégias para aumentar o alcance do público, formado por gestores, jornalistas, profissionais da saúde, pacientes e estudantes da área da saúde, ou seja, um público estratégico, para que receba informações médicas relevantes.

Para os editores científicos dessas revistas assegura-se que os caminhos para aumentar a visibilidade, ampliar o alcance a públicos diversos, com enfoque em estudantes de medicina e profissionais da saúde, no caso da *NEJM*, e influenciar as mudanças nas políticas públicas de saúde, mais fortemente no caso de *The Lancet*, estão na divulgação científica como estratégia eficiente e relevante nas linhas editoriais de ambas, com mais engajamento nas redes sociais e esforços de comunicação para não especialistas realizados pela *NEJM*.

Por fim, vale destacar que há profissionalização e investimento em estratégias de *marketing*, editoração e divulgação, o que faz com essas revistas permaneçam como referências na área da medicina, tanto na acadêmica quanto na mídia. As revistas analisadas angariam enorme interesse público e jornalístico, resultado da visibilidade nas redes sociais e dos esforços de comunicação empenhados.

O engajamento e os investimentos de *The Lancet* e da *NEJM* servem como modelos para revistas de medicina e de outros campos do conhecimento repensarem sua missão na comunicação científica, com a possibilidade de atingir públicos que estejam além dos especialistas de áreas específicas em que atuam, sua responsabilidade social e sua visibilidade e seu prestígio na academia e na sociedade. Apesar dos esforços de comunicação e de controle no fluxo editorial de artigos, as publicações seguem com o desafio de garantir credibilidade, alta qualidade e responsabilidade social dos conteúdos que publicam.

Este estudo se limitou a uma amostra pequena de publicações que continham as missões das revistas, as linhas editoriais, as mudanças e as críticas sobre a relação das revistas com o público e a sociedade, balizadas pelas comemorações de aniversários de *The Lancet* e *NEJM*, sem esgotar o tema. Novos estudos poderão ser realizados lançando mão de análises quantitativas ou de conteúdo para trazer outros aspectos relativos à comunicação pública da medicina.

## Data Availability

https://doi.org/10.25824/redu/BKS5UN.

## NOTAS

1 Em 11 de fevereiro de 2021, o bioRxiv e o medRxiv permitiram o acesso a 11.918 *preprints* sobre covid-19 disponibilizados em 2020.

2 A altmetria, ou métricas complementares, mede e quantifica a atenção social que um artigo recebe em plataformas digitais como *blogs*, Wikipedia, notícias, postagens no Facebook ou X, entre outras.

3 O FI tem sido usado para medir o impacto, o prestígio ou a qualidade de uma revista científica, apesar de inúmeras críticas sobre seu uso.

4 De acordo com dados do *Journal Citation Reports*, compartilhados nos *websites* de cada revista.

5 O Web of Science (WoS) foi escolhido por ser o indexador de literatura internacional com campos de busca mais estruturados para pesquisas em 2018, quando esta pesquisa teve início. Atualmente há outras bases de dados, como Dimensions (2018) e OpenAlex (2022), que poderão futuramente ser usadas para novas análises.

6 Nesta e nas demais citações de textos em inglês a tradução é livre.

7 Elaborado pelo Ministério de Saúde da Inglaterra em 1920 e considerado um dos primeiros documentos a utilizar o conceito de atenção primária à saúde em uma perspectiva de organização sistêmica regionalizada e hierarquizada de serviços de saúde, por nível de complexidade e em uma base geográfica definida.

8 Informações extraídas originalmente do *site* da revista *The Lancet* em julho de 2015, mas atualizadas em agosto de 2025. “Information for authors”. Disponível em: https://www.thelancet.com/lancet/about. Acesso em: 15 ago. 2025.
